# Identification, Structural, and Expression Analyses of *SPX* Genes in Giant Duckweed (*Spirodela polyrhiza*) Reveals Its Role in Response to Low Phosphorus and Nitrogen Stresses

**DOI:** 10.3390/cells11071167

**Published:** 2022-03-30

**Authors:** Jingjing Yang, Xuyao Zhao, Yan Chen, Gaojie Li, Xiaozhe Li, Manli Xia, Zuoliang Sun, Yimeng Chen, Yixian Li, Lunguang Yao, Hongwei Hou

**Affiliations:** 1 The State Key Laboratory of Freshwater Ecology and Biotechnology, The Key Laboratory of Aquatic Biodiversity and Conservation of Chinese Academy of Sciences, Institute of Hydrobiology, Chinese Academy of Sciences, Wuhan 430072, China; yangjj@ihb.ac.cn (J.Y.); zhaoxuyao@ihb.ac.cn (X.Z.); chenyan@ihb.ac.cn (Y.C.); ligaojie@ihb.ac.cn (G.L.); lixiaozhe202109@163.com (X.L.); xiamanli@ihb.ac.cn (M.X.); zlsun@ihb.ac.cn (Z.S.); chenym@ihb.ac.cn (Y.C.); liyixian@ihb.ac.cn (Y.L.); 2 University of Chinese Academy of Sciences, Beijing 100049, China; 3 Henan Key Laboratory of Ecological Security for Water Source Region of Mid-line of South-to-North Diversion Project of Henan Province, Nanyang 473061, China; lunguangyao@163.com; 4 Collaborative Innovation Center of Water Security for Water Source Region of Mid-line of South-to-North Diversion Project of Henan Province, Nanyang Normal University, Nanyang 473061, China

**Keywords:** SPX, *Spirodela polyrhiza*, phosphorus stress, nitrogen stress, gene structure, gene expression profiling

## Abstract

*SPX* genes play important roles in the coordinated utilization of nitrogen (N) and phosphorus (P) in plants. However, a genome-wide analysis of the *SPX* family is still lacking. In this study, the gene structure and phylogenetic relationship of 160 *SPX* genes were systematically analyzed at the genome-wide level. Results revealed that *SPX* genes were highly conserved in plants. All *SPX* genes contained the conserved SPX domain containing motifs 2, 3, 4, and 8. The 160 *SPX* genes were divided into five clades and the *SPX* genes within the same clade shared a similar motif composition. P1BS *cis*–elements showed a high frequency in the promoter region of *SPX*s, indicating that *SPX* genes could interact with the P signal center regulatory gene *Phosphate Starvation Response1* (*PHR1*) in response to low P stress. Other *cis*–elements were also involved in plant development and biotic/abiotic stress, suggesting the functional diversity of *SPX*s. Further studies were conducted on the interaction network of three *SpSPXs*, revealing that these genes could interact with important components of the P signaling network. The expression profiles showed that *SpSPX*s responded sensitively to N and P deficiency stresses, thus playing a key regulatory function in P and N metabolism. Furthermore, the expression of *SpSPXs* under P and N deficiency stresses could be affected by environmental factors such as ABA treatment, osmotic, and LT stresses. Our study suggested that *SpSPXs* could be good candidates for enhancing the uptake ability of *Spirodela polyrhiza* for P nutrients in wastewater. These findings could broaden the understanding of the evolution and biological function of the *SPX* family and offer a foundation to further investigate this family in plants.

## 1. Introduction

Phosphorus (P) is one of the most important nutrients for plant growth and development. It is a component of biofilms, nucleic acids, and ATP, and it participates in many physiological and biochemical processes, such as energy metabolism, signal transduction and photosynthesis [1]. Plants prefer to absorb inorganic phosphate (Pi) to meet their needs for growth [2]. However, the level of Pi accessible to plants is generally low because of their low solubility and mobility in soil [2]. Therefore, low P stress is one of the most important limiting factors for crop yields in addition to nitrogen (N). The application of P fertilizer continues to increase in modern agriculture to achieve high agricultural productivity and meet the demand of the global population for food, leading not only to the eutrophication of water bodies but also the waste and depletion of P [3]. Thus, revealing the mechanism of plant adaptation to a low P environment is of great importance to improve the absorption and utilization of P in plants.

Plants have evolved many adaptive responses to low P stress, such as “Pi starvation responses” (PSRs), to increase the absorption and utilization of P, including the remodeling of root system architecture, synthesis, and secretion of organic acids and phosphatase to promote the release of insoluble Pi in soil, Pi transport and remobilization, reduction in photosynthesis, and starch and anthocyanin biosynthesis in leaves [4]. An MYB transcription factor (TF) called *Phosphate Starvation Response1* (*PHR1*) is the central regulator in the PSRs regulatory system in plants via the transcriptional regulation of *P starvation-induced* (*PSI*) genes involved in the above physiological and biochemical responses. *PHR1* could directly bind to the *cis*-element P1BS (PHR binding sequence, GNATATNC) [5], which is located in the promoter regions of many *PSI* genes, including *Pi transporters* (*PT*), *Phosphate transporter traffic facilitator 1* (*PHF*1), Phosphate 1 (PHO1), *miRNA399*, *miRNA827*, and *IPS1* (*INDUCED BY PHOSPHATE STARVATION1*) [6]. Under low P stress, *PHR1* promotes an increase in Pi uptake by directly inducing the transport of PT and PHF1 from the endoplasmic reticulum to the plasma membrane [7]. Furthermore, *PHR1* indirectly inhibits *Phosphate 2* (*PHO2*) (Target of *miR399*) and *Nitrogen limitation adaptation* (*NLA*) (Target of *miR827*), which mediate post-translational negative control of PT and PHO1 through the ubiquitin-mediated protein degradation pathway, thus increasing the Pi content of the aboveground parts of plants [8,9]. *IPS1* competitively binds to *miR399* to inhibit the degradation of PHO2 [8].

Although *PHR1* could initiate the expression of a series of *PSI* genes under low P conditions, the expression of *PHR1* is barely modulated by external Pi status [10]. Recent studies have reported that SPX proteins harboring only the well-conserved N-terminal hydrophilic SPX domain (Pfam PF03105) are negative regulators that substantially suppress PHR1 activity or translocation. However, as Pi itself does not bind to the SPX domain, the inositol pyrophosphate InsP_8_ is the intracellular Pi signaling molecule serving as the ligand of SPX1 for controlling Pi homeostasis in plants, with levels positively correlated with cellular Pi concentration [11].The SPX domain was named after the SYG1 (suppressor of yeast gpa1), the PHO81 (yeast cyclin-dependent kinase inhibitor), and the XPR1 (human xenotropic and polytropic retrovirus receptor 1), which was first identified in the yeast *Saccharomyces cerevisiae* [12]. In plants, the SPX domain-containing proteins are classified into four subfamilies depending on the nature of the C-terminal extra domains, including SPX, SPX–EXS (named after the yeast ERD1, human XPR1 and Yeast SYG1 proteins), SPX–Major Facility Superfamily (SPX–MFS), and SPX–Really Interesting New Gene (SPX–RING) subfamilies [12]. Many SPX domain–containing proteins, such as PHT5 (SPX–MFS) [13], PHO1 (SPX–EXS) [14], and AtNLA (SPX–RING) [9] have been proven to be widely involved in Pi signaling and Pi balance.

In Arabidopsis (*Arabidopsis thaliana*) and rice (*Oryza sativa*), four (*AtSPX1*–*4*) and six members (*OsSPX1*–*6*) of *SPX* genes have been identified, respectively. Except for *SPX4*, the expression of *AtSPX1*–*3*, *OsSPX1*–*3*, and *OsSPX5*–*6* is induced by low P stress with distinct dynamic patterns. AtSPX1/2 are located in the nucleus, and they bind to AtPHR1, forming a stable protein complex when the P content is sufficient, thus preventing the binding of *AtPHR1* to P1BS elements of *PSI* genes [15,16]. Under low P conditions, the expression of *AtSPX1/2* in Arabidopsis leaves and roots is strongly and continuously induced. *AtPHR1* is released by *AtSPX1/2* and then binds to *PSI* genes, thus inducing low P responses [15,16]. *OsSPX1/2* regulate *OsPHR2* through a similar pathway as *AtSPX1/2* in response to low P stress [17,18]. *OsSPX3/5* are expressed in leaves and roots induced by low P, and they negatively regulate the transport of P from underground to aboveground parts [19]. OsSPX4/6 are located in the nucleus and cytoplasm. OsSPX4/6 interact with OsPHR2 in the cytoplasm and partially inhibit the transfer of OsPHR2 into the nucleus, thereby inhibiting P signaling. When P is sufficient, OsSPX4 maintains a high abundance and mainly inhibits OsPHR2. Under low P stress, the expression of *OsSPX6* is significantly enhanced at the transcriptional level; however, at the protein level, OsSPX4/6 are degraded by the ubiquitination–26S proteasome pathway; thus, OsPHR2 is released and activates low Pi responses [18,20]. Recent studies have found that *OsSPX4* participates in the regulation pathway of N and P signals centered at *OsNLP3* (NIN–like protein) and *OsPHR2* to promote the synergistic absorption and utilization of N and P in rice [21].

Duckweeds are aquatic monocotyledonous plants belonging to the family Lemnaceae, which contains five genera (*Spirodela*, *Landoltia*, *Lemna*, *Wolffiella*, and *Wolffia*) and 36 species in total, and they are widely distributed in various fresh habits around the world [22]. Interest in duckweeds has steadily increased over the past decade, driven by the growing need for duckweeds as an alternative for traditional agricultural crops that can help tackle urgent societal challenges, such as climate change and rapid population expansion [23]. Duckweeds have traditionally been used as human food source, animal feed, feedstocks for biofuel and biogas production, and the production of proteins products [23]. Among them, the giant duckweed (*Spirodela polyrhiza*) is the biggest species with the smallest genome size of approximately 158 Mbp [24]. At present, the genomic and transcriptome data of *S. polyrhiza* are abundant [24]. Moreover, a stable and efficient genetic transformation system has been established; therefore, *S. polyrhiza* plants are regarded as a model for duckweed plants [25]. *S. polyrhiza* plants could easily cultivate and propagate new buds rapidly, doubling the biomass by 1.34–4.54 days [26]. *S. polyrhiza* plants had a very high uptake ability for Pi nutrients in wastewater and could adapt to a Pi concentration range of 0.1–46.5 mg/L [27]. Their rapid growth quickly converts the absorbed nutrients into starch biomass [26]. Therefore, *S. polyrhiza* plants are the most studied and applied aquatic plants in wastewater treatment [28]. However, the Pi content in most eutrophic water is 0.05–0.09 mg/L, far lower than the demand of duckweed plants [27]. This fact limits the application of *S. polyrhiza* plants in the treatment of eutrophic water. Therefore, developing *S. polyrhiza* plants adapting to a wider range of P concentrations based on their own mechanism of adaptation to low P stress is of great significance.

The functions of *SPX* genes have been comprehensively analyzed in the model of Arabidopsis plants [15] and rice [29,30], and in some important crops, including soyabean (*Glycine max*) [31], rapeseed (*Brassica napus*) [32], and wheat (*Triticum aestivum*) [33], which revealed that SPX genes are key regulators in the P signal regulatory network [34]. SPX genes may be valuable targets for improving the absorption and utilization of P and enhancing tolerance to low P stress in plants. In the present paper, a genome-wide identification and characterization analysis of *SPX* members in yeast, algae, ferns, moss, and some important monocot and dicot plants was conducted. The chromosomal localization, gene duplication, molecular interaction networks, homology modeling, and expression profiles of *SPX* genes in giant duckweed (*S. polyrhiza*) were further analyzed to reveal its role in response to low P stress. This study provides a solid foundation for further functional characterization of *SPX* genes in a wide range of plant species, especially in duckweeds.

## 2. Materials and Methods

### 2.1. Identification of SPX Genes

Genome databases of *A. thaliana, Amborella trichopoda, Brachypodium distachyon, B. napus, Carica papaya, Chlamydomonas reinhardtii, Citrus clementine, Eucalyptus grandis, G. max, Gossypium hirsutum, Ipomoea triloba, Nelumbo nucifera, O. sativa, Physcomitrella patens, Populus trichocarpa, Prunus persica, Ricinus communis, S. cerevisiae, Salvinia cucullata, Selaginella moellendorffii, Setaria italic, Solanum lycopersicum, S. polyrhiza, T. aestivum, Wolffia australiana, Zea mays*, and *Zostera marina* were downloaded from NCBI database (https://www.ncbi.nlm.nih.gov/, accessed on 8 January 2021), Phytozome database (https://phytozome.jgi.doe.gov/pz/, accessed on 11 February 2021) and Ensembl plant database (https://plants.ensembl.org/index.html, accessed on 20 February 2021). The genome database of *Azolla filiculoides* was downloaded from Fernbase (https://www.fernbase.org/, accessed on 12 February 2021).

The Hidden Markov Model (HMM) profile of the SPX domain (PF03105) was downloaded from the Pfam database (http://pfam.xfam.org/, accessed on 15 March 2021). All the non-redundant *SPX* genes sequences of *A. thaliana*, *O. sativa*, and *G. max* were downloaded from the NCBI website and used as query sequences to search for *SPX* genes in the other 23 species by BLASTP with a threshold of 1e < −10. The *SPX* genes of *B. napus* and *T. aestivum* have been published elsewhere [32,33] The HMMER v 3.3.2 program (http://hmmer.org/, accessed on 20 March 2021) was also used to identify *SPX* genes [35]. Putative *SPX* genes were further confirmed by the SMART database (http://smart.embl-heidelberg.de/, accessed on 6 April 2021), NCBI Conserved Domain database (http://www.ncbi.nlm.nih.gov/Structure/cdd/wrpsb.cgi, accessed on 6 April 2021), and Pfam database.

### 2.2. Physiochemical Properties, Subcellular Localization and SSRs (Simple Sequence Repeats) Identification

The number of amino acids, molecular weight (MW), and isoelectric point (PI) of the SPX proteins were calculated using the online ExPASy-ProtParam (http://web.expasy.org/protparam/, accessed on 21 April 2021) [36]. The transmembrane helices (TMHs) of the SPX proteins were predicted using the online TMHMM Server v.2.0 (http://www.cbs.dtu.dk/services/TMHMM/, accessed on 28 April 2021) [37]. The subcellular localization of all SPX proteins was predicted by Cello v2.5 software (http://cello.life.nctu.edu.tw/, accessed on 28 April 2021) [38], PSORT (https://psort.hgc.jp/, accessed on 28 April 2021) [39], and BUSCA (http://busca.biocomp.unibo.it, accessed on 28 April 2021) [40]. The SSRs in 160 *SPX* genes were identified by MISA software (https://webblast.ipk-gatersleben.de/misa/, accessed on 5 March 2022) [41]. The minimum number of repetitions was set as five for mononucleotide SSR detection, three for dinucleotide SSRs, and two for trinucleotide to decanucleotide. The maximum length of sequence between two SSRs was set at 100, which was registered as compound SSR.

### 2.3. Phylogenetic Classification, Gene Structures, and Motif Analysis

All the identified 160 *SPX* genes were aligned by ClustalW [42]. A maximum likelihood phylogenetic tree was constructed by MEGA 7.0 with 1000 bootstrap replications [43] and then visualized by iTOl (https://itol.embl.de/, accessed on 26 February 2021) [44]. The Gene Structure Display Server 2.0 online program (GSDS 2.0, http://gsds.gao–lab.org/, accessed on 20 March 2021) was used to analyze the exon–intron structure information of all *SPX* genes [45]. The MEME online program was used to identify the conserved motifs in SPX proteins (https://meme-suite.org/meme/tools/meme, accessed on 28 March 2021) with the following parameters: any number of repetitions; maximum number of motifs, 10; and optimum motif length, 6–50 [46].

### 2.4. Cis-Acting Elements, Chromosomal Localization, Gene Duplication, and Synteny Analysis

The 2000 bp DNA sequence upstream (promoter sequence) of the start codon of all *SPX* genes was extracted by TBtools software (V1.9832.0.0) [47]. The *cis*-acting elements of the promoter sequence were analyzed by the New Place online program (https://www.dna.affrc.go.jp/PLACE/?action=newplace, accessed on 22 April 2021) and visualized using TBtools. Synteny and gene duplication were analyzed by MCScanX and MCScanX–transposed [48,49]. The chromosomal localization of *SPX* genes on *S. polyrhiza* was determined by TBtools on the basis of genome annotation gff3 file. The syntenic relationships among nine species, including *A. thaliana*, *B. distachyon*, *G. max*, *O. sativa*, *P. trichocarpa*, *S. italic*, *S. lycopersicum*, *S. polyrhiza*, and *Z. mays*, were identified by MCScanX and visualized by Tbtools [48,50].

### 2.5. Molecular Interaction Networks and Protein Structure Prediction for SpSPX Proteins

The STITCH v5.0 server (http://stitch.embl.de/, accessed on 3 May 2021) was used to construct an interaction network between SpSPX proteins and other proteins on the basis of the Arabidopsis genome [51]. The 3D structures of three SpSPX proteins were predicted by the trRosetta (transform-restrained Rosetta) server, a new web-based platform for fast and accurate protein structure prediction, powered by deep learning (https://robetta.bakerlab.org/, accessed on 15 March 2022) [52,53,54]. Deep learning is now becoming an indispensable component for improving the accuracy of protein structure prediction, rather than the homology modeling method on the basis of the resolved structure of homologous proteins [52,53,54].

### 2.6. Expression Profiles of SpSPX Genes under Low P/N Stress

#### 2.6.1. Expression Analysis of SpSPX Genes by RNA-Seq Data

First, RNA-seq was used to explore the expression profiles of *SpSPX* genes under low P/N stress (Bio–Project PRJNA724886). *S. polyrhiza* 7498 plants were cultured in liquid half-strength (1/2) MS medium at pH 5.8 and under 25 ± 1 °C, an irradiance of 85 μmol photons PAR m^−2^ s^−1^, and a 16 h photoperiod [55]. Then, *Spirodela* plants were cultured with 1/2 MS medium [+P/+N, the control group (CK)], 1/2 MS medium with N deficiency (+P/−N, N deficiency group), 1/2 MS medium with P deficiency (−P/+N, P deficiency group), 1/2 MS medium without N and P (−P/−N, N–P deficiency group), and H_2_O (nutrient deficiency group) for 7 day. All the plants in different treatments were harvested at 7 day and immediately frozen in liquid N and then stored at −80 °C for RNA isolation. Experiments were performed in triplicate to allow statistical analyses of the results. The expression profile data of *SpSPX* genes in different tissues/organs (leaves, roots, and stipule; Bio–Project PRJNA557001) and under different concentrations of salt stress (Bio–Project PRJNA563960) were also obtained from the NCBI website. The data was statistically analyzed using SPSS 22.0 software. One-way analysis of variance (ANOVA) and the Duncan test were applied to determine the significance of differences among different groups at the *p* < 0.05 level. All the Sequence Read Archive accession numbers are displayed in Table S1.

#### 2.6.2. Expression Analysis of SpSPX Genes by qRT–PCR (Quantitative Real-Time PCR)

qRT–PCR was used to further analyze the expression of *SpSPX* genes under low P/N stress. *S. polyrhiza* 7498 plants were first pre-cultured in liquid 1/2 MS medium at pH 5.8, under 25 ± 1 °C, an irradiance of 85 μmol photons PAR m^−2^ s^−1^, and a 16-h photoperiod (CK) [55]. Then, they were treated with 1/2 MS medium with P deficiency (−P) and N deficiency (−N) and supplemented with 1 μM KH_2_PO_4_ and NH_4_NO_3_ for 10 day. The treated plants were harvested at set time points (0 [CK], 1, 2, 3, 4, 5, 6, 7, and 10 day) and immediately frozen in liquid N and then stored at −80 °C for RNA isolation with three biological replicates.

To investigate whether the expression of *SpSPX* genes was regulated by abiotic stresses, we analyzed the expression of *SpSPX* genes in some abiotic stress conditions including abscisic acid (ABA) treatment (1 µM), osmotic stress (200 mM mannitol), and low temperature (LT, 10 °C) stress under low P/N conditions. The *S. polyrhiza* 7498 plants were cultured in liquid 1/2 MS medium (1/2 MS), 1/2 MS medium with P (-P) or N (-N) deficiency containing 1 µM ABA or 200 mM mannitol at pH 5.8, and under 25 ± 1 °C, an irradiance of 85 μmol photons PAR m^−2^ s^−1^, and a 16-h photoperiod. Similarly, for LT treatment, *S. polyrhiza* 7498 plants were cultured in liquid 1/2 MS medium (1/2 MS), 1/2 MS medium with P (-P) or N (-N) deficiency at pH 5.8, and under 10 ± 1 °C, an irradiance of 85 μmol photons PAR m^−2^ s^−1^, and a 16-h photoperiod. To investigate whether the expression of *SpSPX* genes was correlated with some transcription factors (TFs), we further analyzed the expression of *SpWRKY75* and MYB-domain TF *SpNIGT1.1* (*NITRATE-INDUCIBLE GARP-TYPE TRANSCRIPTIONAL REPRESSOR 1.1*) under LT and low P/N stress conditions. The *WRKY75* was the first member of the WRKY TF family reported to be involved in regulating P starvation response [56]. The NIGT1.1 protein has been reported to modulate P starvation signalling via transcriptional regulation of *SPX* genes [57]. The treated plants were harvested at set time points (0 [CK], 1, 3, 5, and 7 day) and immediately frozen in liquid N and then stored at −80 °C for RNA isolation with three biological replicates.

The total RNA of *S. polyrhiza* 7498 plants was extracted using a commercial RNA extraction kit (CW2598S, CWBIO, Beijing, China). First-strand cDNA was synthesized using a PrimeScript™ RT Reagent Kit (TaKaRa, Dalian, China). TB Green^®^ Premix Ex Taq™ (TaKaRa, Dalian, China) was used to perform qRT−PCR with the Bio–Rad CFX96 Touch Real time PCR System (Bio-Rad, Hercules, CA, USA). The qRT–PCR program was as follows: 10 min at 95 °C, 40 cycles of 95 °C for 15 s, 60 °C for 30 s, and 72 °C for 30 s; a melting process at 60–95 °C was carried out to generate the melting curve. The *18s* gene was used as an internal control. Each reaction was analyzed in triplicate, and the 2^−ΔΔCT^ method was used to analyze the data [58]. The qRT–PCR results were statistically analyzed according to methods mentioned in Chapter 2.6.1. The oligonucleotide primers of three *SpSPX*s, *SpWRKY75,* and *SpNIGT1.1* are shown in Table S2.

## 3. Results

### 3.1. Identification and Phylogenetic Analysis of SPX Genes in 28 Species

First, HMMER and BLASTP were used for the identification of *SPX* genes from 28 species. Then, the genes were further confirmed by Pfam and CDD online websites. Ultimately, 160 *SPX* genes with only SPX domains were identified in the 28 species (Table 1). These *SPX* genes were named in accordance with the order of their gene locations in chromosomes or scaffolds. The *SPX* genes of *A. thaliana*, *O. sativa*, and *G. max* have been named in previous studies. Only one *SPX* gene was identified in yeast while algae *S. cerevisiae* had nine *SPX* genes. The ancient moss plant *P. patens* had seven *SPX* genes. However, no *SPX* gene was found in the moss *Marchantia polymorpha*. The fern plants *A. filiculoides* and *S. cucullata* had four *SPX* genes. The fern plant *S. moellendorffii* had six *SPX* genes. The number of *SPX* genes among the monocot species ranged from 2 to 15. Compared with monocot species, the dicot species had 3 to 12 *SPX* genes. The maximum number of 15 *SPX* genes was identified in the monocot species *T. aestivum*, followed by the dicot species *G. hirsutum* (12 in total)*, B. napus* (11 in total), and *G. max* (nine in total). The amino acid length of 160 *SPX* genes ranged from 184 to 491; correspondingly, the molecular weight ranged from 3.4654 KDa to 54.712 KDa, the isoelectric point (PI) ranged from 4.68 to 10.28, and the number of exons between 1 and 16. However, none of the 160 SPX proteins had TMHs (Table S3). Most of the SPX proteins were located in the nucleus and cytoplasm, while some were located in the chloroplast, mitochondrion, and plasma membrane (Table S3). We identified a total of 7018 SSRs in 160 *SPX* genes (Table 2). In 10 types of SSR, mononucleotide, dinucleotide, and tetranucleotide SSRs are dominant with cumulative percentages of 35.95%, 23.25%, and 17.63%, respectively, whereas hept- to hexanucleotide SSRs are rare with 0.07–0.94%. Almost all the *SPX* genes contained mononucleotide to pentanucleotide SSRs, while only *NnSPX2*, *NnSPX*3, *GhSPX6*, *OsSPX1*, and *SlSPX6* contained decanucleotide. The (A/T)n, (AG/CT)n (AG/CT)n, (AAG/CTT)n, and (AAAG/CTTT)n were the most frequent motifs in mononucleotide, dinucleotide, trinucleotide, and tetranucleotide SSRs. The number of compound SSRs ranged from 3 (*TaSPX10*) to 246 (*NnSPX2*) in 160 *SPX* genes. The detailed numbers of SSRs in 160 *SPX* genes were shown in Table S4.

**Figure 1:**
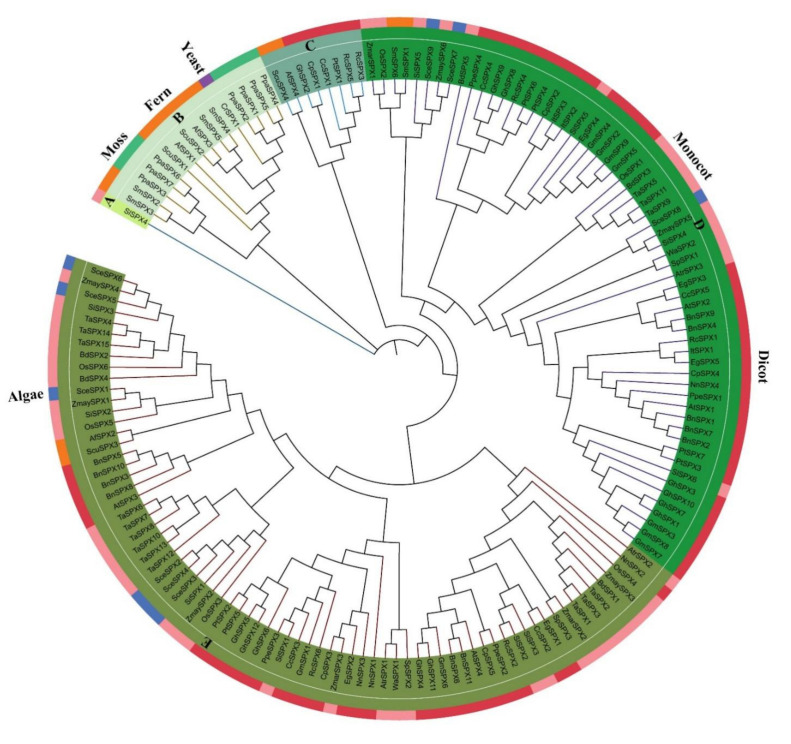
Phylogenetic analysis of 160 *SPX* genes in 28 species, including yeast, algae, moss, fern, monocot, and dicot plants. Maximum likelihood tree was generated by MEGA 7.0 with 1000 bootstrap replications on the basis of LG model. The 160 *SPX* genes were divided into five groups and labeled by different colors.

The genes were classified by phylogenetic analyses on the basis of the maximum likelihood method to understand the evolutionary relationships of the *SPX* genes. The 160 *SPX* genes were divided into five clades (A–E), as shown in Figure 1. The member proportion of each clade was different. Clade E (46.25%) had the most genes, followed by clade D (38.125%), clade B (10%), and clade C (5%). The *SlSPX4* of *S. lycopersicum* formed a single clade away from other clades. Almost all the genes in moss and yeast were clustered in clade B. The *SPX* genes of fern plants were distributed in four clades (B, C, D, E). The *SPX* genes of algae, which were closely related to *ZmaySPXs*, were only distributed in clades D and E. Furthermore, clades D and E contained the most dicot and monocot plant species. The *ScuSPX4*, *AfSPX4*, and some *SPX* genes of dicot species were clustered in the second small clade C.

### 3.2. Gene Structure and Conserved Motifs of SPX Genes

**Figure 2:**
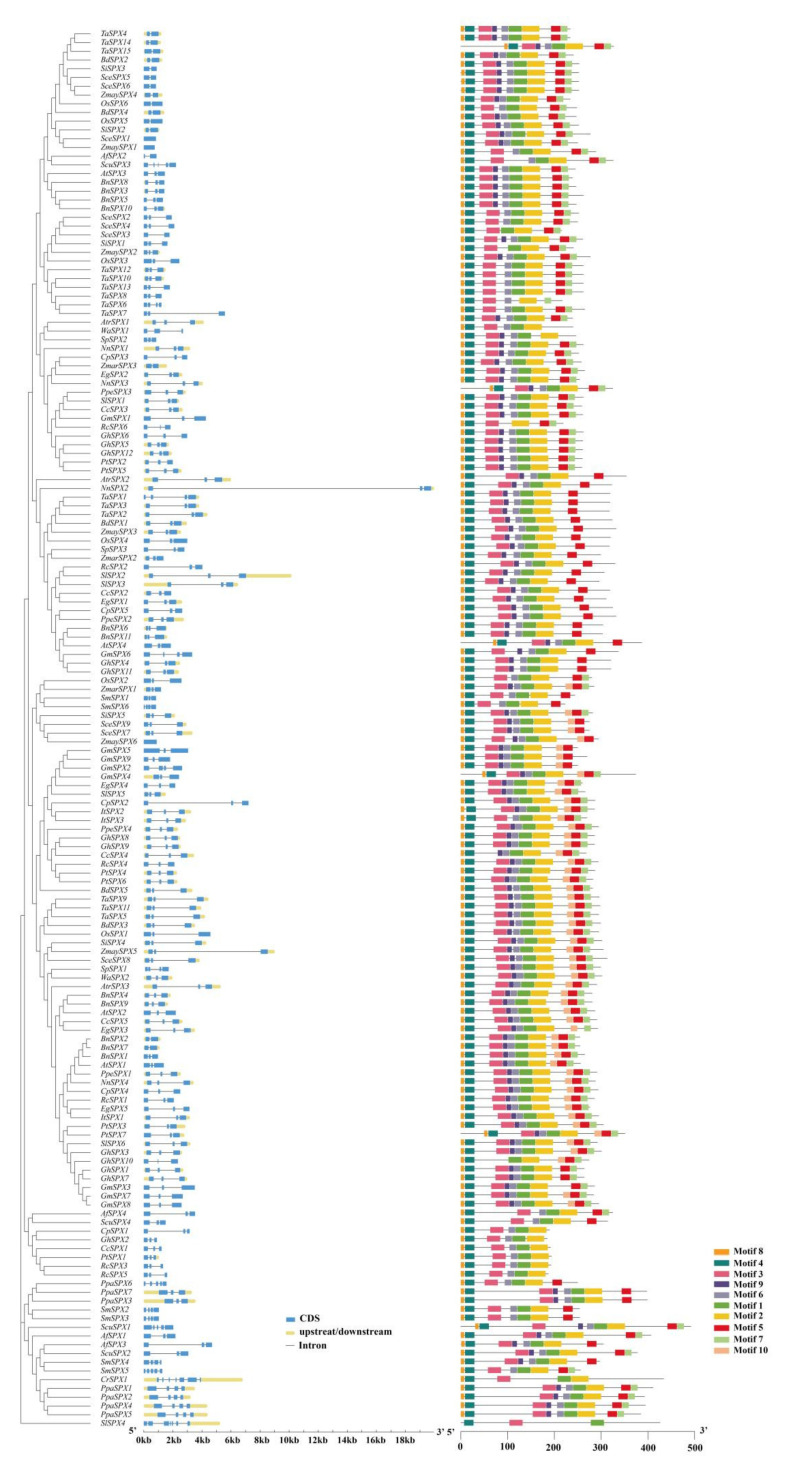
*Cis*-elements and conserved domain motifs in 160 *SPX* genes.

The GSDS online server was used to determine the gene structures of all *SPX* genes. As shown in Figure 2, most genes on the same branch of the phylogenetic tree had similar numbers of introns and exons. *NnSPX2* had the largest length (approximately 20 kb), with only three exons and two introns. *ZmaySPX1* had a minimum length (755 bp), and it contained one coding region. Clade A (*SlSPX4*) had seven exons and six introns, while it was three exons for other *SlSPX* genes. The genes in clade B had 3–7 exons and 2–6 introns. However, all the genes in clade C contained only three exons and two introns. Most genes in clades D and E also had three exons and two introns. The schematic structures of all *SPX* genes were constructed using the MEME analysis tool. The obtained sequence and length information of the conserved motif are shown in Figure 2 and Table S5. All *SPX* genes contained the conserved SPX domain containing motifs 2, 3, 4, and 8. The *SPX* genes within the same clade shared a similar motif composition but showed distinct differences in motif composition among different clades. For example, the genes in clade C contained motifs 1, 2, 3, 4, 6, and 8. In clade D, these genes contained almost all 10 motifs, while most genes in clade E lacked motifs 7 and 10. The genes in clade B contained motifs 8, 4, 3, 6, 1, 2, and 5, and clade A only consisted of motifs 1, 3, and 4. In total, the similar gene structures and conserved motif compositions of the *SPX* genes within the same clade strongly supported the reliability of the phylogenetic analysis results.

### 3.3. Cis-Regulatory Element Analysis of SPX Genes

**Figure 3:**
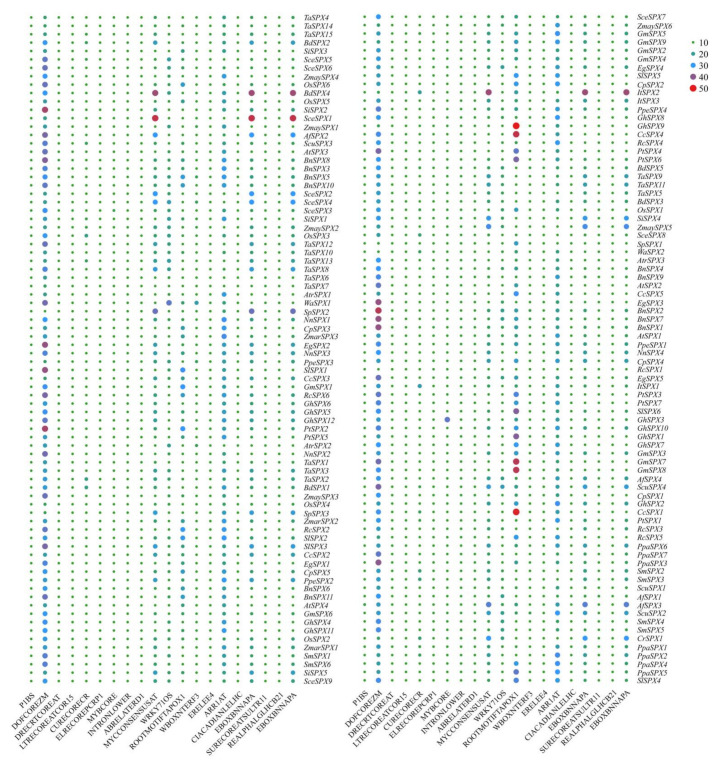
Frequency of main *cis*-elements in the 2 kb promoter region of 160 *SPX* genes.

The 2 kb upstream regions of *SPX* genes of 28 species were extracted to analyze the *cis*–acting elements using the online software NEWPLACE to investigate the potential function and regulatory mechanisms of *SPX* genes (Figure 3). Most of the *SPX* genes contained 2, 4, 6, or 8 P1BS *cis*–elements, consistent with the previous reports [32,33,59]. This finding suggested that these *SPX* genes could be bound by *PHR* transcription factors involved in response to low P stress. In Arabidopsis and rice, *PHR1* acted on many *PSI* genes by preferentially binding to *cis*–element P1BS in the promoters of these genes to activate the low P response and promote Pi uptake [5,20,60]. Other *cis*–elements were also found to be involved in development (DOFCOREZM, ROOTMOTIFTAPOX1), tissue-specific expression (EBOXBNNAPA, L1BOXATPDF1), dehydration responses (DRECRTCOREAT), low–temperature responses (TRECOREATCOR15), plant hormone responses (ABRELATERD1, ERELEE4, ARR1AT), light responses (CIACADIANLELHC, REALPHALGLHCB21, EVENINGAT), copper ion-induced expression elements (CURECORE–CR), injury response elements (WBOXNTERF3), and other kinds of biotic/abiotic stress. Some *cis*-elements usually bound by MYB, MYC, and WRKY TFs, such as MYBCORE, MYCCONSENSUSAT, and WRKY71OS, were also identified, indicating that the expression of *SPX* genes might be regulated by various MYB, MYC, and WRKY TFs when the external Pi concentration changes. Among these *cis*–elements, the DOFCOREZM and ROOTMOTIFTAPOX1 elements were the most abundant, followed by the ARR1AT, EBOXBNNAPA, MYCCONSENSUSAT, and CURECORECR elements.

### 3.4. Chromosomal Localization, Gene Duplication and Synteny Analysis of SPX Genes in S. polyrhiza

**Figure 4:**
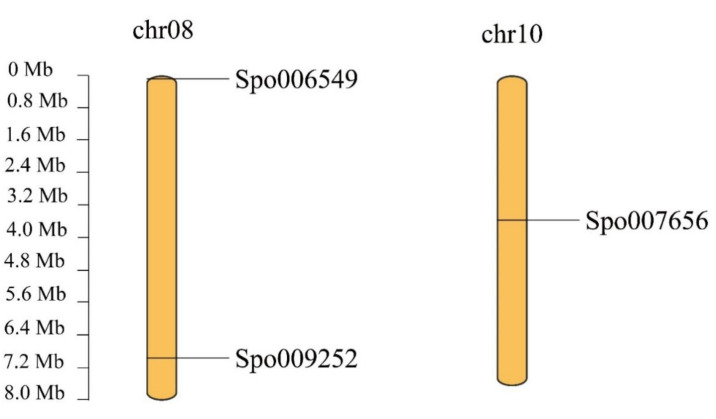
Distribution and synteny relationship of *SpSPX* genes on the chromosomes of *S. polyrhiza*. The chromosomes were indicated by orange color. *SpSPX1* (Spo006549) and *SpSPX3* (Spo009252) were located at chromosome 08 (chr08), and *SpSPX2* (Spo007656) was located at chromosome 10 (chr10). Scale bar indicated chromosome length (Mb).

**Figure 5:**
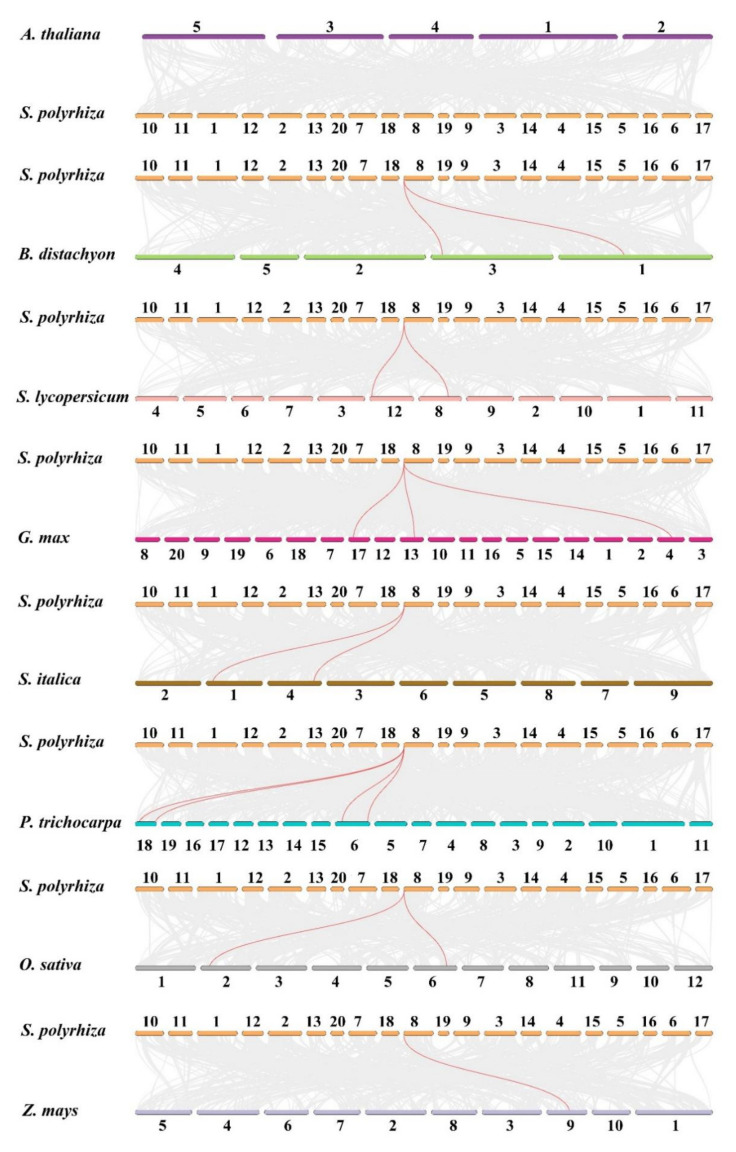
Synteny relationship of *SPX* genes pairs between *S. polyrhiza* and other seven plant species. Red represents syntenic *SPX* genes, and gray lines show the collinear blocks of the plant genome. The chromosome number was labeled at the top of each chromosome.

Synteny analysis was conducted at the chromosome-level genome assembly of the duckweed species of *S. polyrhiza* via McScan software. The results showed that no tandem repeat or segmental duplication events occurred in *S. polyrhiza*. In addition, three *SPX* genes were identified in *S. polyrhiza*, and their chromosome distributions were analyzed. *SpSPX1* (Spo006549) and *SpSPX3* (Spo009252) were located at chromosome number 8 (chr08), and *SpSPX2* (Spo007656 *SpSPX3*) was located at chromosome number 10 (chr10) (Figure 4). The evolutionary relationships of the *SPX* family between *S. polyrhiza* and eight other species, including *A. thaliana* (Brassicaceae), *B. distachyon* (Poaceae), *S. lycopersicum* (Solanaceae), *G. max* (Fabaceae*)*, *S. italic* (Poaceae), *P. trichocarpa* (Salicaceae), *O*. *sativa* (Poaceae), and *Z. mays* (Poaceae), were further analyzed. A collinearity gene map of the *SPX* family among these nine species is shown in Figure 5 and Table 3. No syntenic *SPX* gene pair between *A. thaliana* and *S. polyrhiza* could be found in the map. Moreover, 1–4 syntenic *SPX* gene pairs between *S. polyrhiza* and seven other species could be observed. Only *SpSPX1* was found to be collinear with the *SPX* genes of other species. Four syntenic *SPX* gene pairs were identified between *S. polyrhiza* and *P. trichocarpa* (on chr06 and chr18). Two syntenic *SPX* gene pairs could be found between *S. polyrhiza* and *B. distachyon* (on chr01 and chr03)*, S. lycopersicum* (chr08 and chr12)*, S. italic* (chr01 and chr04), and *O. sativ*a (chr02 and chr06). Only one syntenic *SPX* gene pair was found between *S. polyrhiza* and Z. may (chr09).

### 3.5. Molecular Interaction Networks and Protein Structure Prediction for SpSPXs

**Figure 6:**
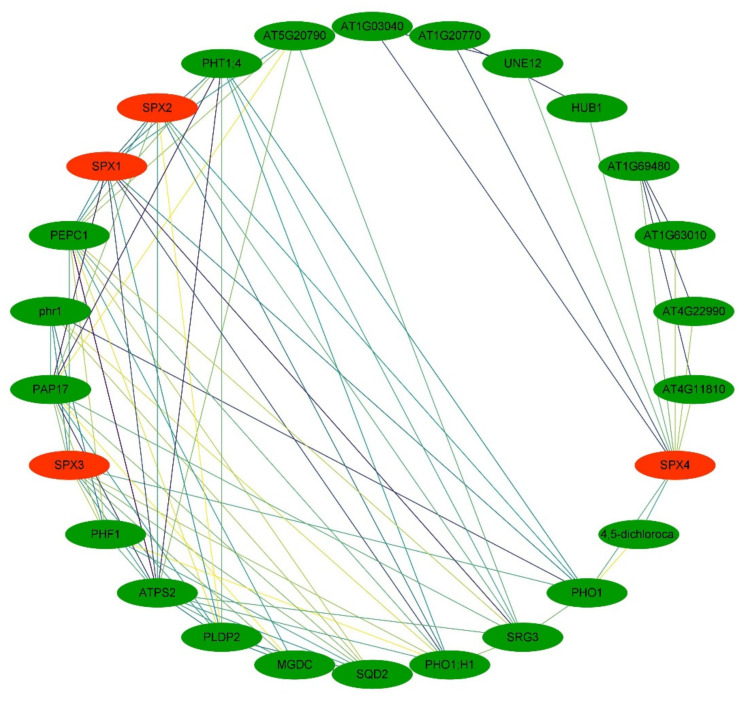
Predicted interaction network of *SpSPX* genes and their targets according to Arabidopsis. The interacted genes were connected by different lines.

**Figure 7:**
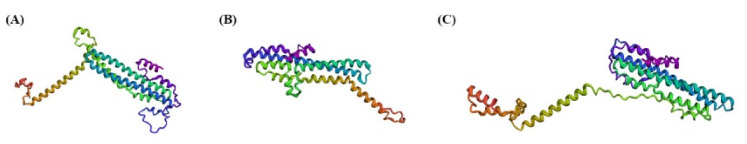
(**A**–**C**) The predicted 3D structure of three SpSPX proteins by trRobetta server, (**A**)-SpSPX1 protein, (**B**)-SpSPX2 protein, and (**C**)-SpSPX3 protein.

An interaction network between *SpSPXs* and their targets was constructed on the basis of the Arabidopsis genome (Figure 6). The results of the interaction network involved four *SPX* genes and 22 targets. Most of the targets were involved in low P response and P metabolism, including *PHR* (the central regulator in PSR regulatory system), *Phosphate starvation-induced gene 2* (*ATPS2*), Pi transporters (*PHO1*, *PHO1; H1*, and *PHT1; 4*), *Purple acid phosphatase 17* (*PAP1*7) that is involved in the activation and utilization of Pi, PEPC1 (Pyridoxal phosphate phosphatase-related protein) that catalyzed the cleavage of pyrophosphate, Phospholipase D P2 (PLDP2) that is involved in regulating root development in response to Pi limitation, SPX–MFS proteins (AT4G11810, AT4G22990, and AT1G63010), and SPX–EXS protein (AT1G69480). The *Senescence–related gene 3* (*SRG3*) was also identified to interact with *SpSPX1* and *SpSPX2*. Some TFs, such as *UNE12,* which is required for ovule fertilization, and AT1G03040 (TF bHLH7 identifier), which regulates the competence of pericycle cells to initiate lateral root primordium formation, were found in the interaction network of *SpSPXs*. The interaction network contained some enzymes, transmembrane proteins, and macromolecular compounds, such as AT5G20790 (transmembrane protein); AT1G20770 (coiled–coil protein); and monogalactosyldiacylglycerol synthase 3 (MGDC), which is involved in the synthesis of the major structural component of photosynthetic membranes and in chloroplast envelope biogenesis, sulfoquinovosyldiacylglycerol 2 (SQD2), and 4, 5–dichlorocatechol. The 3D structures of three SpSPX proteins predicted by the trRosetta server are shown in Figure 7A–C. The modeled 3D structure of three SPX proteins consisted of two parts, including “SPX domain” and “non-SPX domain”. The “SPX domain” parts of three SPX proteins shared a high level of homology, thus providing information to explore their functions at the proteomic level.

### 3.6. Transcriptional Patterns of SpSPX Genes in S. polyrhiza

**Figure 8:**
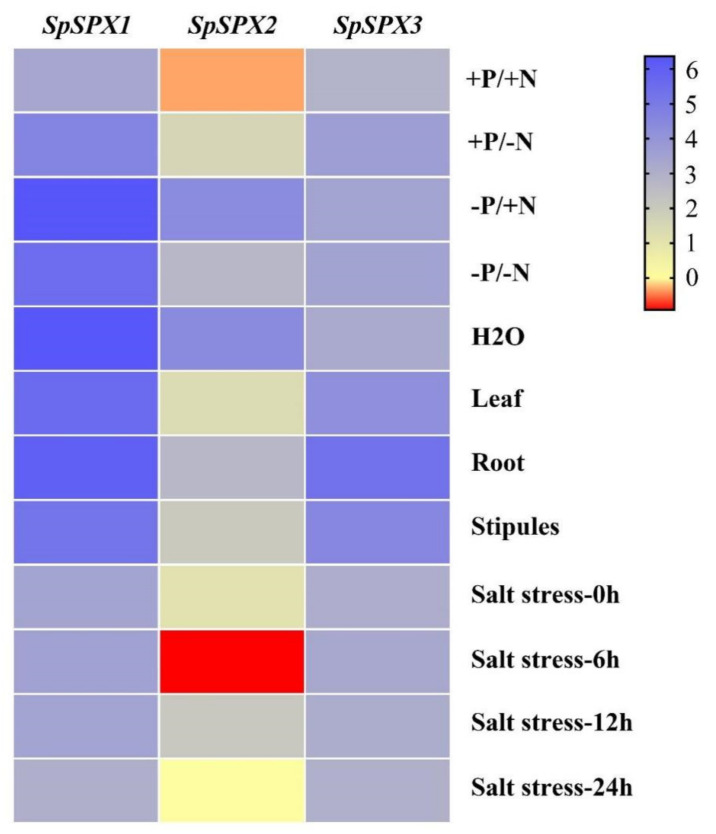
Expression profiles of *SpSPX* genes under abiotic stress conditions (P/N stress, and salt stress) and in different tissues (leaf, root, and stipules).

RNA–seq was used to explore the expression profiles of three *SpSPX* genes under low P/N stress (Figure 8). The expression profiles *of SpSPX* genes were shown as log2 values on the basis of TPM values (transcripts per kilobase of exon model per million mapped reads). The expression of *SpSPX1* in the N deficiency, P deficiency, N–P deficiency, and nutrient deficiency groups increased to high levels compared with that in the CK group, especially in the P deficiency and nutrient deficiency groups. *SpSPX2* expression only increased in the P deficiency and nutrient deficiency groups. The expression levels of *SpSPX3* under different treatments were almost the same as those in the CK group. The above results indicated that *SpSPX1* was more sensitive to N and P stresses than *SpSPX2* and *SpSPX3*, which could rapidly respond to low P/N stresses and regulate the expression of other interacting genes. The expression of *SpSPXs* in previous studies was also analyzed, including different plant tissues and under salt stress treatment (Figure 8). The results showed that *SpSPX1* was highly expressed in roots, leaves (mature fronds/mother fronds), and stipules (young fronds/daughter fronds). However, the expression of *SpSPX2* in roots, leaves, and stipules was relatively low, and the expression levels were similar among various tissues. The expression of *SpSPX3* in different tissues was higher than that of *SpSPX2* and only at a high level in roots. The results suggested that the expression of these *SpSPX* genes had a tissue-specific pattern. However, the heatmap revealed that the expression of *SpSPX* genes was not affected by salt stress or the time of treatment.

### 3.7. Expression Analysis of SpSPX Genes in Response to low P/N Stress by qRT-PCR

**Figure 9:**
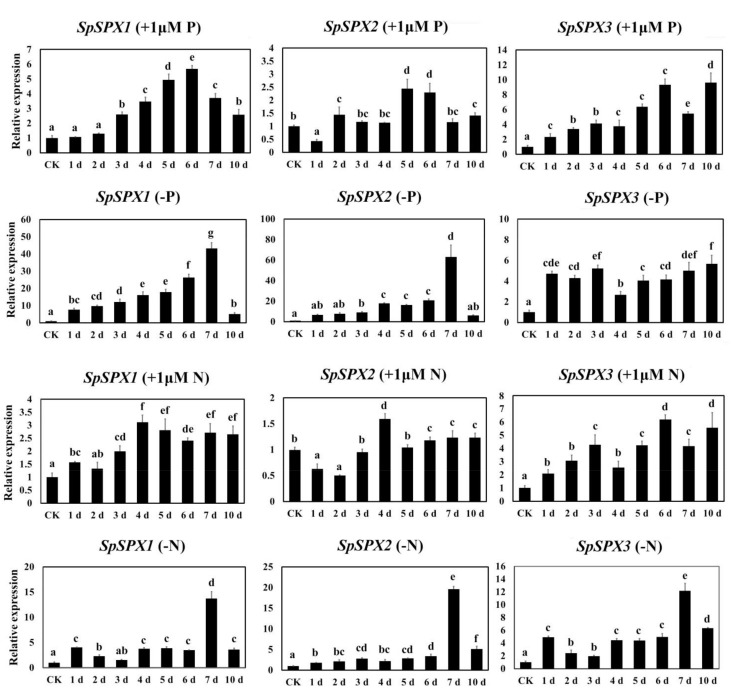
Quantitative RT-PCR analysis of three *SpSPX* genes under 1 μM P/N and P/N deficiency conditions from 1 day to 10 day. Different letters indicate significant differences between treatments at the *p* < 0.05 level (Duncan test). Error bars indicate standard deviation.

The expression of three *SpSPX* genes under different P/N stresses was further analyzed by qRT–PCR (Figure 9). When treated with 1μM P, *SpSPX1* significantly upregulated from 1 day to 6 day and then downregulated at 7 day and 10 day. However, no significant difference was observed between the CK group and the 1 day and 2 day groups. By contrast, the expression of *SpSPX1* significantly increased from 1 day to 7 day and was reduced to its lowest level at 10 day under P deficiency. A significant difference was observed between the CK group and the 1d group. Furthermore, the expression of *SpSPX1* under P deficiency was significantly higher than that under 1 μM P treatment. Similarly, the expression of *SpSPX1* under N deficiency was significantly higher than that under 1 μM N treatment. Although the expression of *SpSPX1* from 1 day to 10 day under N deficiency was significantly higher than that of the CK group, the highest expression of *SpSPX1* was observed at 7 day. The expression of *SpSPX1* was significantly upregulated from 1 day to 4 day and then downregulated from 5 day to 10 day, with no significant difference. The expression of *SpSPX2* was significantly downregulated at 1 day and then upregulated at 2, 5, and 6 day by 1 μM P treatment. No significant difference was found among the CK, 3, 4, 7, and 10 day groups. The expression of *SpSPX2* was significantly downregulated at 1 day and 2 day by 1 μM P treatment and then upregulated from 3 day to 6 day. The highest expression of *SpSPX2* was observed at 4 d, and its expression was maintained at a stable level from 6 d to 10 d. The expression changes of *SpSPX2* were similar under P and N deficiency, significantly upregulated from 1 day to 7 day, and significantly downregulated at 10 day. The expression of *SpSPX2* under P/N deficiency was higher than that under 1 μM P/N. All the expression levels of *SpSPX3* were significantly upregulated at 1 day by all treatments. The highest expression of *SpSPX3* was observed at 10 day under 1 μM P and P deficiency, 6 day under 1 μM N, and 7 day under P deficiency. The expression of *SpSPX3* was significantly upregulated from 1 day to 6 day by 1 μM P treatment, significantly downregulated at 7 day, and increased to its highest level at 10 day. All the expression levels of *SpSPX3* from 1 day to 10 day were relatively higher than those in the CK group under P deficiency. However, no significant difference was observed between 1 day and 2 day, 1 day and 3 day, 5 day and 6 day, 6 day and 7 day, and 7 day and 10 day. Although downregulated from 3 day to 4 day and 6 day to 7 day, the expression of *SpSPX3* was significantly upregulated by 1 μM N treatments from 1 day to 10 day compared with that of the CK group. The expression of *SpSPX3* was also significantly upregulated from 1 day to 10 day under N deficiency. These results suggested that *SpSPX* genes were induced by low P/N stresses and played critical roles in the regulation of P/N responses.

**Figure 10:**
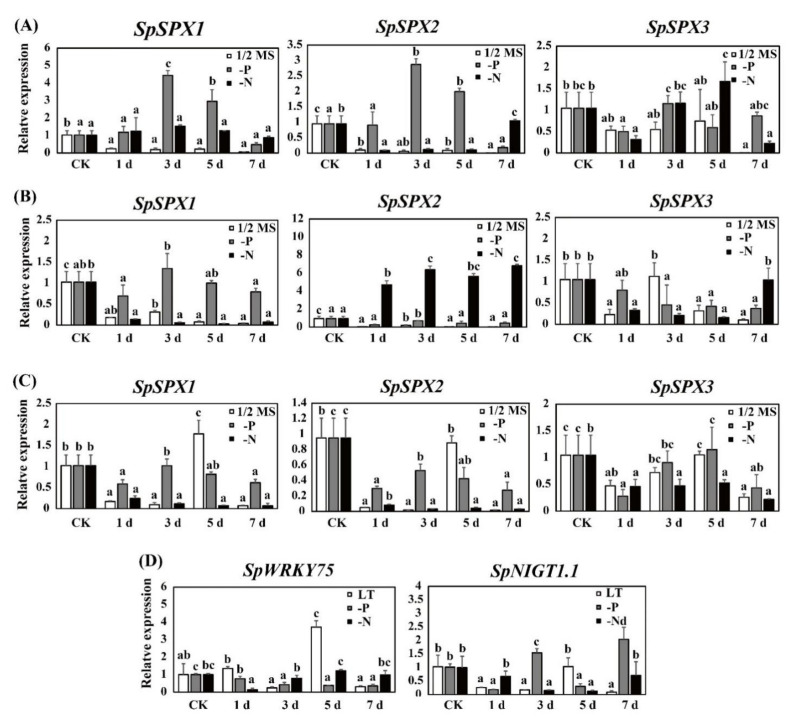
Quantitative RT-PCR analysis of three *SpSPX* genes under ABA treatment (**A**), osmotic stress (**B**), and LT stress (**C**) in 1/2 MS medium and 1/2 MS medium with P/N deficiency (-P, -N) at 0 [CK], 1, 3, 5, 7 d. Quantitative RT-PCR analysis of *SpWRKY75* and *SpNIGT1.1* under LT stress (10 °C) and low P/N stresses at 0 [CK], 1, 3, 5, 7 d (**D**). Different letters indicate significant difference between treatments at the *p* < 0.05 level (Duncan test). Error bars indicate standard deviation.

The expression of three *SpSPX* genes was also regulated by ABA treatment, osmotic, and LT stresses (Figure 10A–C). When *Spirodela* plants were cultured in 1/2 MS medium, all the expressions of three *SpSPX* genes were downregulated at 1 day under these stress conditions. Furthermore, the expression of *SpSPX1 and SpSPX2* was significantly downregulated from 1 day to 7 day when treated by ABA and osmotic stresses. The expression of *SpSPX1 and SpSPX2* was significantly upregulated from 1 day to 3 day and then downregulated to the lowest at 7 day when treated by LT stress. The expression levels of *SpSPX3* were upregulated from 1 day to 5 day and downregulated to the lowest at 7 day when treated by ABA and LT stresses. When treated by osmotic stress, the expression of *SpSPX3* was significantly upregulated from 1 day to 3 day and then downregulated from 5 day to 7 day. When *Spirodela* plants were cultured in 1/2 MS medium with P and N deficiency, the expression of *SpSPX*1 was upregulated from 1 d to 5 day and then downregulated at 7 day by ABA treatment. Similarly, the expression of *SpSPX2* was also upregulated from 1 day to 5 day at 1/2 MS–P group under ABA treatment and from 1 day to 7 day at 1/2 MS -N group under osmotic stress. However, all the expression levels of three *SpSPX* genes were downregulated at 1 day in the remaining treatments. Furthermore, the expression levels of the *SpSPX1* at 1/2 MS-N group under osmotic and LT stresses, the *SpSPX2/3* at 1/2 MS-N group under LT stress, and the *SpSPX3* at 1/2 MS-P group under osmotic stress were downregulated from 1 day to 7 day. By contrast, almost all the expression levels of *SpSPXs* in the remaining treatments were upregulated from 1 day to 3 day. The above results indicated that ABA treatment, osmotic and LT stresses inhibited the expression of *SpSPXs* even when *Spirodela* plants were cultured in P and N deficiency conditions. Although the expressions of *SpSPXs* were upregulated in the late treatment, such as 3 day or 5 day, the expression of *SpSPXs* was maintained at a relatively low level compared to those in plants without ABA, osmotic stress, and LT stress.

The expressions of TFs *SpWRKY75* and *SpNIGT1.1* under LT and low P/N stress conditions are shown in Figure 10D. When treated by LT stress, the expression of *SpWRKY75* was significantly upregulated at 1 d. The highest expression of *SpWRKY75* was observed at 5 day, and then significantly downregulated at 7 day. The expression of *SpWRKY75* was contrary to that of *SpSPXs*, with levels significantly downregulated from 1 day to 7 day by P deficiency treatment. By contrast, the expression of *SpWRKY75* was reduced to its lowest level at 1 day and then significantly increased from 3 day to 7 day by N deficiency treatment, which was similar to the expression of *SpSPXs*. The expression of *SpNIGT1.1* was significantly downregulated from 1 day to 3 day and then significantly upregulated at 5 day by LT stress. The lowest level of *SpNIGT1.1* was observed at 7 day. The expression of *SpNIGT1.1* was significantly downregulated from 1 day to day by N deficiency treatment and then significantly increased to the highest level at 7 day, which was contrary to that of *SpSPXs* under N deficiency condition. However, the expression of *SpNIGT1.1* was only significantly downregulated at 1 day and 3 day by P deficiency treatment. The expression levels of *SpNIGT1.1* at 3 day and 7 day were significantly higher than that in CK group.

## 4. Discussion

Whole-genome sequencing of many plants has been completed thanks to the rapid development of high-throughput sequencing technology, which provides the convenience to carry out gene family analysis [61]. The *SPX* genes play an important role in the P signal network, including Pi absorption, transport, storage, and homeostasis [32]. However, studies on the identification and functional analysis of *SPX* genes involved in the P signal network in aquatic plants have not yet been reported. In the present investigation, 160 *SPX* genes were identified in the genomes of 28 species. The results revealed that the monocot species *T. aestivum* and dicot species *G. hirsutum* had more *SPX* genes than algae, ancient moss, and fern plants, possibly due to a higher abundance of intrachromosomal gene duplications [62]. The number ranges of *SPX* genes between monocot and dicot plants were close. However, the basal angiosperm *A. trichopoda* had only three *SPX* genes. These results indicated that *SPX* genes also played a similarly important functional role in ancestral species and that gene expansion occurred in the evolution from ancestral species to monocot and dicot plants. Although seven *SPX* genes were identified in the ancient moss plant *P. patens*, no *SPX* gene was found in the moss *M. polymorpha.* These results indicated that not only gene retention occurred during the evolution of plants but also gene loss [59]. The numbers of *SPX* genes in terrestrial plants were much higher than those in aquatic plants, such as *S. polyrhiza*, *W. australiana*, and *Z. marina*. This finding may be due to the difference in the distribution and content of P nutrients between terrestrial and aquatic environments, thus affecting the acquisition and absorption of P nutrients by plants [63]. Through phylogenetic tree analysis, these 160 *SPX* genes were divided into five clades. This was further supported by the results of gene structure and conserved motifs. The *SPX* genes in these five clades exhibited gene length and exon-intron structural divergences. However, the exon-intron and conserved motif structures of *SPX* genes were highly conserved within each clade, and the genes clustered together generally possessed a similar distribution of intronic regions amid the exonic sequences. For example, *SlSPX4* clearly formed a single clade away from other clades, possibly due to its different gene structure and motif composition. *SlSPX4* mainly consisted of three motifs (motifs 1, 3, and 4), fewer than genes in other clades. Moreover, *SlSPX4* contained six introns and seven exons. However, the *SPX* genes in clade B were mostly composed of motifs 1–9 with four introns and five exons, while the *SPX* genes in clade C were only composed of motifs 1–4, 6, and 8 with two introns and three exons. By contrast, the *SPX* genes in clade D had almost all 10 motifs and contained two introns and three exons. Most of the *SPX* genes in clade E had motifs 1–9, while some genes, such as *SceSPX1* and *ZmaySPX1*, contained only one intron or had no introns. The different motif compositions and exon–intron structures may contribute to the functional diversification of *SPX* genes.

The *cis*–elements in promoter regions play important roles in gene regulation and expression during plant growth and responses to diverse biotic and abiotic stresses [64]. Therefore, investigating *cis*–elements is necessary for the identification of genes related to plant development or stress resistance [65]. In response to low P stress, master transcriptional activator *PHR1* acted on many *PSI* genes by preferentially binding to *cis*-element P1BS, thus activating a low P response and promoting Pi uptake [5,20,60]. In Arabidopsis and rice, *AtSPX1/2* or *OsSPX1/2* acted as negative regulators, inhibited *OsPHR2/AtPHR1* activity through direct protein–protein interactions, and prevented the binding of *PHR2* (or *AtPHR1*) to the promoter regions of *PSI* genes in a Pi-dependent manner [16,17]. The present study identified that most *SPX* genes contained two, four, six, or eight P1BS *cis*-elements, consistent with previous reports and thus suggesting that these *SPX* genes could be bound by low P-responsive transcriptional activator *PHR1* in response to a low P response [16,17,20,66]. However, their response to P signaling and regulatory ability to the *PHR1* gene may be different due to the difference in the number of P1BS elements in different *SPX* genes. In addition, other *cis*-elements involved in plant development and biotic/abiotic stress, such as dehydration responses, low-temperature responses, plant hormone responses, light responses, and injury response elements, and some TFs, such as MYB and WRKY, were identified; this finding indicated that *SPX* genes also participate in other important life activities in addition to Pi absorption and transport in plants, consistent with the findings in Arabidopsis [66,67]. Our previous study also reported that *SpWRKY* genes rapidly responded to P deficiency at 2 h treatment and 12 *SpWRKYs* were differentially expressed under P nutrients stress [55]. However, research on the diverse functions of *SPX* genes in other species is lacking.

The chromosomal localization, gene duplication, and synteny analysis of *SPX* genes in *S. polyrhiza*, the largest duckweed plant that has a strong enrichment ability for P/N, were further analyzed to explore the evolution and functions of *SPX* genes in aquatic plants [27]. In the present study, no tandem repeat or segmental duplication events occurred in *S. polyrhiza*, possibly because of its primitive evolutionary position, small genome size, and relatively simple genome structure [24]. The three *SPX* genes were identified in *S. polyrhiza*, with chromosome distributions at chr08 (*SpSPX1* and *SpSPX2*) and chr10 (*SpSPX3*), which minimized the possibility for gene duplication events. The evolutionary relationships of the *SPX* family between *S. polyrhiza* and eight other species were also analyzed. Many colinear gene pairs among these species were revealed. Furthermore, only *SpSPX1* was collinear with the *SPX* genes in other species, indicating that *SpSPX1* was more conserved than *SpSPX2* and *SpSPX3*. Two syntenic *SPX* gene pairs were found between *S. polyrhiza* and rice, and 1–4 syntenic *SPX* gene pairs were found between *S. polyrhiza* and other species, indicating that the *SPX* genes were highly conserved among these species. However, no syntenic *SPX* gene pair was identified between *A. thaliana* and *S. polyrhiza* because of their distant relationship.

The interaction network between SpSPX proteins and other proteins also provides basic information for further functional characterization of SpSPX proteins. The analysis results showed that most of the targets of *SpSPXs* were involved in P dynamics, including *PHR1*, *ATPS2*, PHO1, PHO1; H1, PHT1; 4, PAP17, PEPC1, PLDP2, SPX–MFS proteins, and SPX–EXS protein, which have been confirmed to interact with SPX proteins in Arabidopsis and rice [6]. Yan et al. (2019) characterized *AtSPX1* as a key regulator in mediating the crosstalk among leaf senescence, Pi starvation, and salicylic acid (SA) signaling pathways in Arabidopsis [68]. The present study also identified that SpSPX1 and SpSPX2 proteins could interact with *SRG3*, indicating that the SpSPX proteins played an important role in plant senescence induced by low P stress. The interaction between SpSPX1 and SpSPX2 was also identified, implying their functional redundancy, and these two proteins may form homodimers and heterodimers in response to P starvation. In rice, *SPX3/5* were verified to be functional repressors of *OsPHR2* involved in restoring Pi balance with similar tissue expression patterns and subcellular localization. These results suggested that the evolution of the additional redundant paralogous *SPX* genes was beneficial to plants recovering Pi homeostasis after Pi starvation by the *PHR2* pathway [19]. The interaction network also contained some TFs, enzymes, transmembrane proteins, macromolecular compounds, SQD2, and 4, 5–dichlorocatechol. In soybean, *GmSPX1* was proven to interact with *GmMYB48* and possibly function as a negative regulator of *AtMYB4*, an ortholog of *GmMYB48* under low Pi conditions [31]. Ruan et al. (2018) reported that *OsSPX1* interacted directly with *REGULATOR OF LEAF INCLINATION1* (*RLI1*) to prevent *RLI1* binding to the promoters of its downstream genes, *BU1* (*BRASSINOSTEROID UPREGULATED1*), and *BU1-LIKE 1 COMPLEX1*, to control elongation of lamina joint cells, thereby enhancing leaf inclination [69]. Two RING-finger ubiquitin E3 ligases, SDEL1 and SDEL2, facilitated the degradation of OsSPX4 to modulate *PHR2* activity and regulate Pi homeostasis and Pi signaling in response to external Pi availability in rice [70]. These results indicated the functional diversity of *SpSPX* genes and their importance in plant development, stress response, and other life processes.

In aquatic environments, N and P stresses are the main restrictions on the growth of aquatic plants, thereby affecting their adaptability to environmental changes [55]. In the present study, the transcriptional pattern profiles of three *SpSPX* genes under low P/N stress were analyzed. The results showed that *SpSPX1* was highly expressed by low P/N stresses compared with *SpSPX2* and *SpSPX3*. Moreover, *SpSPX1* and *SpSPX2* were more sensitive to low P stress than to low N stress, with relatively higher expression levels. Furthermore, *SpSPX1* was also highly expressed in all the tissues of *S. polyrhiza* plants. *SpSPX2* and *SpSPX3* had a higher expression in roots than in leaves and stipules. This finding suggested that *SpSPX1* and *SpSPX3* mainly played a key regulatory role in root tissue. Different expression levels of the *SPX* gene in shoots and roots were also found in rice [18]. *OsSPX1* and *OsSPX2* had relatively higher expression in shoots, while *OsSPX3*, *OsSPX5*, and *OsSPX6* were highly expressed in roots [18]. By contrast, the three *SpSPX* genes did not highly respond to salt stress, suggesting that *SpSPX* genes may not be involved in salt stress response. This finding was consistent with the above results, in which no salt stress-related *cis*-elements or interacting proteins were identified. However, our qRT–PCR results revealed that three *SpSPX* genes are sensitive to ABA treatment, osmotic, and LT stresses. These stresses inhibited the expression of *SpSPXs* even when *Spirodela* plants were cultured in P and N deficiency conditions, indicating that the transcriptional regulation of N and P responses by *SpSPXs* was affected by external environmental factors (ABA, osmotic, and LT stresses). We speculate that these environmental stresses may affect the acquisition of N and P nutrients, therefore inhibiting the response of *SpSPXs* to low N and P stresses. However, the underlying mechanism is still worthy of in-depth study. These results also confirmed our *cis*-element analysis results because the relevant *cis*-elements are found in the promoter regions of three *SpSPX* genes. The qRT–PCR results of *SpSPXs* low N and P stresses also verified the transcriptome data. The expression of *SpSPX1*, *SpSPX2*, and *SpSPX3* was highly induced by low N and P stresses, and their transcripts continually accumulated to very high levels with prolonged treatments. The expression of *SpSPXs* under P stress was similar to that in Arabidopsis, rice, and soybean [15,18,31]. However, the expression of *GmSPXs* was still maintained at high levels at 10 d under P stress [31], while *SpSPX1* and *SpSPX2* was significantly downregulated at 10 d under P/N deficiency. These facts suggested that *SpSPXs* expression was highly induced by low P/N conditions, thereby playing a key regulatory function in P and N metabolism. Although some key genes related to N metabolism were not identified in the above interaction protein analysis, some previous studies have reported the mechanism underlying the balanced acquisition of N and P regulated by SPX proteins. For example, the NIGT1.1 (SPX–PHR cascade-mediated N status)-responsive regulation of Pi uptake and starvation signals in Arabidopsis [57]. Furthermore, the NRT1.1–SPX4 interaction promoted OsSPX4 ubiquitination and degradation via E3 ubiquitin ligase, which in turn led to the enhancement of Pi acquisition via activation of OsPHR2 [21,71]. In current study, we also analyzed the expression of *SpNIGT1.1* under LT stress and P and N deficiency conditions. Results showed that the expression of *SpNIGT1.1* was inhibited by these stresses at 1 d, which was opposite to the expression of *SpSPXs* under N and P deficiency conditions. However, the expression of *SpNIGT1.1* continually accumulated to high levels with prolonged treatments under LT and P deficiency conditions. These results indicated that *SpNIGT1.1*-mediated regulation has generally antagonistic effects on the expression of *SpSPXs.* Similar results have been reported in Arabidopsis that N-induced expression of *NIGT1.1* modified the expression level of *SPX* genes by severely reduced the activity of *SPXs* promoters [57]. The WRKY family is one of the largest TF families in plants and plays vital roles in various biotic and abiotic processes [72]. In our previous study [55], we analyzed the expression of 47 *SpWRKY* genes under abiotic stress including P deficiency stress, cold stress, and submergence stress [55]. We found that the *SpWRKY* genes rapidly responded to the P deficiency stress while the expression of *SpWRKYs* involved in cold and submergence stresses changed gradually over time. In current study, we further analyzed the expression of *SpSPX75* (named *SpSPX10* in [55]. Our results are consistent with Zhao et al., (2021) in that the expression of *SpSPX75* was upregulated in response to cold stress at 1 d and downregulated from 1 d to 7 d by P deficiency stress [55]. Furthermore, the expression of *SpSPX75* was upregulated from 1 d to 7 d by N deficiency stress, which was similar to the expression patterns of *SpSPXs*. However, Devaiah et al., (2007) reported that the expression of *WRKY75* was strongly induced upon Pi starvation and was rapidly suppressed by Pi resupply. These results indicated that the regulation function of *WRKY75* is diverse and *WRKY75* responds in a different way to P and N deficiency stresses [56]. Considering the critical role of *SPX* genes in the regulation of P and N starvation signals, determining their functions in *S. polyrhiza* is important to provide a foundation for the development of duckweed strains with low P/N tolerance and high P/N enrichment for alleviating environmental problems, such as water eutrophication.

## 5. Conclusions

*SPX* genes are important regulators in the P/N signal regulatory network. In this study, comprehensive genome-wide identification and structural and expression analyses of *SPX* genes in 28 species were performed. A total of 160 *SPX* genes were characterized and divided into five clades, with high similarity in gene structure and motif composition within the same subfamily. All the *SPX* genes contained at least two P1BS elements and some elements involved in plant development and biotic/abiotic stress. Three *SpSPX* genes were further analyzed, revealing that most of the targets of *SpSPXs* were involved in P metabolism. Expression analysis of *SpSPXs* suggested that *SpSPXs* was highly induced by low P/N conditions, thereby playing a key regulatory function in P and N metabolism. Our findings could broaden our understanding of the evolution and biological function of the SPX family and offer a foundation to further investigate the SPX family in plants.

## Figures and Tables

**Table 1:** Number and type of *SPX* genes in 28 species.

Species	Type of Organism	Group A	Group B	Group C	Group D	Group E	Total
*C. reinhardtii*	Yeast		1				1
*S. cerevisiae*	Algae				3	6	9
*P. patens*	Moss		7				7
*A.filiculoides*	Fern		2	1		1	4
*S. cucullata*	Fern		2	1		1	4
*S. moellendorffii*	Fern		4		2		6
*B. distachyon*	Monocot				2	3	5
*N. nucifera*	Monocot				1	3	4
*O. sativa*	Monocot				2	4	6
*S. italic*	Monocot				2	3	5
*S. polyrhiza*	Monocot				1	2	3
*T. aestivum*	Monocot				3	12	15
*W. australiana*	Monocot				1	1	2
*Z. mays*	Monocot				2	4	6
*Z. marina*	Monocot				1	2	3
*A. thaliana*	Dicot				2	2	4
*A. trichopoda*	Dicot				1	2	3
*B. napus*	Dicot				5	6	11
*C. papaya*	Dicot			1	2	2	5
*C. clementine*	Dicot			1	2	2	5
*E. grandis*	Dicot				3	2	5
*G. max*	Dicot				7	2	9
*G. hirsutum*	Dicot			1	6	5	12
*I. triloba*	Dicot				3		3
*P. trichocarpa*	Dicot			1	4	2	7
*P. persica*	Dicot				2	2	4
*R. communis*	Dicot			2	2	2	6
*S. lycopersicum*	Dicot	1			2	3	6
Total		1	16	8	61	74	160
		0.625%	10%	5%	38.125%	46.25%	

**Table 2:** The distribution of SSRs types in 160 *SPX* genes.

SSR Motif Length	Number	Percentage/%
1	2523	35.95
2	1237	17.63
3	610	8.69
4	1632	23.25
5	602	8.58
6	309	4.40
7	66	0.94
8	23	0.33
9	11	0.16
10	5	0.07

**Table 3:** Identified colinear gene pairs between *S. polyrhiza* and other seven plant species.

Species	Species	Colinear Gene Pairs
*A. thaliana*	*S. polyrhiza*	*-*
*S. polyrhiza*	*B. distachyon*	*SpSPX1*, *BdSPX3*, *BdSPX5*
*S. polyrhiza*	*S. lycopersicum*	*SpSPX1*, *SlSPX5*, *SlSPX6*
*S. polyrhiza*	*G. max*	*SpSPX1*, *GmSPX3*, *GmSPX7*, *GmSPX8*
*S. polyrhiza*	*S. italic*	*SpSPX1*, *SiSPX5*, *SiSPX4*
*S. polyrhiza*	*P. trichocarpa*	*SpSPX1*, *PtSPX3*, *PtSPX4*, *PtSPX6*, *PtSPX7*
*S. polyrhiza*	*O. sativa*	*SpSPX1*, *OsSPX2*, *OsSPX1*
*S. polyrhiza*	*Z. mays*	*SpSPX1*, *ZmaySPX5*

## Data Availability

The datasets used and/or analyzed during the current study are available from the corresponding author on reasonable request.

## Supplementary Materials

The following supporting information can be downloaded at: https://www.mdpi.com/article/10.3390/cells11071167/s1, Table S1: Sequence Read Archive (SRA) accession numbers in S. polyrhiza. Table S2: The oligonucleotide primers of three *SpSPX*s, *SpWRKY75* and *SpNIGT1.1* used for qRT-PCR. Table S3: Physiochemical properties and subcellular localizations of 160 SPX proteins. Table S4: The detailed numbers of SSRs in 160 *SPXs*. Table S5: List of putative motifs of SPX proteins.
