# Supplementary material for: Identification, Structural, and Expression Analyses of SPX Genes in Giant Duckweed (Spirodela polyrhiza) Reveals Its Role in Response to Low Phosphorus and Nitrogen Stresses
Source: Cells. 2022 Mar 30;11(7):1167. doi: 10.3390/cells11071167 (PMC8997716; doi:10.3390/cells11071167)
Supplement: Supplementary file 1 [file cells-11-01167-s001.zip › cells-1579442-supplementary/Table S1-.pdf]

Table S1 The Sequence Read Archive accession (SRA) numbers in *S. polyrhiza*.

Bio-Project	Tissues	Treatments	SRA
PRJNA724886	Whole plant	-	SRR14319996
	Whole plant	-	SRR14319995
	Whole plant	-	SRR14319989
	Whole plant	N-deprivate-7d	SRR14319988
	Whole plant	N-deprivate-7d	SRR14319987
	Whole plant	N-deprivate-7d	SRR14319986
	Whole plant	Nutrient-starvation-7d	SRR14319992
	Whole plant	Nutrient-starvation-7d	SRR14319991
	Whole plant	Nutrient-starvation-7d	SRR14319990
	Whole plant	P/N-deprivate-7d	SRR14319982
	Whole plant	P/N-deprivate-7d	SRR14319994
	Whole plant	P/N-deprivate-7d	SRR14319993
	Whole plant	P-deprivate-7d	SRR14319985
	Whole plant	P-deprivate-7d	SRR14319984
	Whole plant	P-deprivate-7d	SRR14319983
PRJNA557001	Leaves	-	SRR9849994
	Leaves	-	SRR9849993
	Leaves	-	SRR9849996
	Roots	-	SRR9849995
	Roots	-	SRR9849998
	Roots	-	SRR9849997
	Stipules	-	SRR9850000
	Stipules	-	SRR9849999
	Stipules	-	SRR9849992
PRJNA563960	Whole plant	Salt stress-0h	SRR10070165
	Whole plant	Salt stress-0h	SRR10070164
	Whole plant	Salt stress-0h	SRR10070161
	Whole plant	Salt stress-6h	SRR10070160
	Whole plant	Salt stress-6h	SRR10070159
	Whole plant	Salt stress-6h	SRR10070158
	Whole plant	Salt stress-12h	SRR10070157
	Whole plant	Salt stress-12h	SRR10070156
	Whole plant	Salt stress-12h	SRR10070155
	Whole plant	Salt stress-24h	SRR10070154
	Whole plant	Salt stress-24h	SRR10070163
	Whole plant	Salt stress-24h	SRR10070162
