# Supplementary material for: Identification, Structural, and Expression Analyses of SPX Genes in Giant Duckweed (Spirodela polyrhiza) Reveals Its Role in Response to Low Phosphorus and Nitrogen Stresses
Source: Cells. 2022 Mar 30;11(7):1167. doi: 10.3390/cells11071167 (PMC8997716; doi:10.3390/cells11071167)
Supplement: Supplementary file 1 [file cells-11-01167-s001.zip › cells-1579442-supplementary/Table S2-.pdf]

Table S2 The oligonucleotide primers of three *SpSPXs*, *SpWRKY75* and *SpNIGT1.1* used for qRT-PCR.

Gene name	Primers (5'to 3')
SpSPX1-F	ACACCTTCTTCGTGGAGCAA
SpSPX1-R	TTCACCAGGCCAGTGTAGTTG
SpSPX2-F	GGCTAAAGCGGCAGATCGAG
SpSPX2-R	ATGCGGACGAACTCCAACAC
SpSPX3-F	CAAGTCCATCCCTGTGCCTG
SpSPX3-R	CTCCTGAAGGCGTATGACGAA
SpWRKY75-F	GGCTGTGAAGAATAATAGGTTCCCAAG
SpWRKY75-R	GGTGTGCGTTCCTTCGTAGGT
SpNIGT1.1-F	GCCACCGCCAAGAGCAGATTA
SpNIGT1.1-R	GCCGAGAAGTCTCATCATCCTCAT
18S-F	AAAGTTGGGGGCTCGAAGAC
18S-R	AAGTTTCAGCCTTGCGACCA
