# Supplementary material for: Identification, Structural, and Expression Analyses of SPX Genes in Giant Duckweed (Spirodela polyrhiza) Reveals Its Role in Response to Low Phosphorus and Nitrogen Stresses
Source: Cells. 2022 Mar 30;11(7):1167. doi: 10.3390/cells11071167 (PMC8997716; doi:10.3390/cells11071167)
Supplement: Supplementary file 1 [file cells-11-01167-s001.zip › cells-1579442-supplementary/Table S5- .pdf]

Table S2 List of the putative motifs of SPX proteins.

Motif	Logo	Best Possible Match	E-Value	Width
1	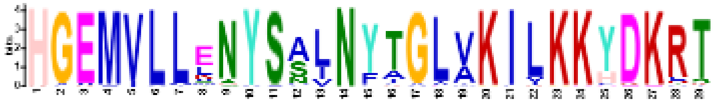	HGEMVLLENYSALNYTGLVKILKKYDKRT	4.0e-4049	29
2	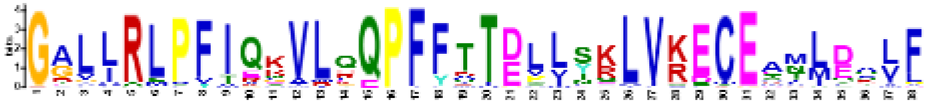	GALIRLPFIQRVLQQPFFTTDLLYKLVKECEAMLDHLF	2.5e-3811	38
3	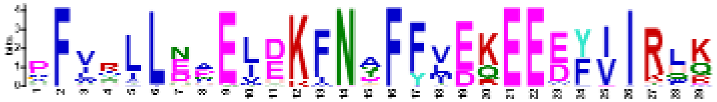	EFVYLLNNEIDKFNAFFMEQEEDFIIRHK	4.4e-2834	29
4	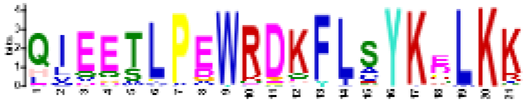	QIEETLPEWRDKFLSYKDLKK	2.5e-2368	21
5	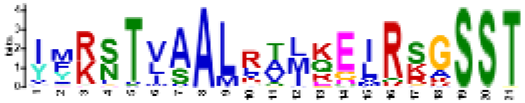	IFRNTVAALLTMQEIRKGSST	1.5e-1857	21
6	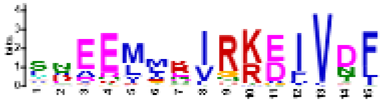	SNEEMMKIRKEIVDF	1.6e-837	15
7	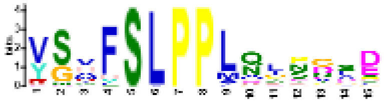	YSHFSLPPLNLPDSD	3.6e-688	15

8	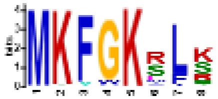	MKFGKWLK	1.3e-660	8
9	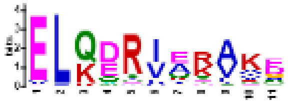	ELQDRVAKAKD	2.7e-347	11
10	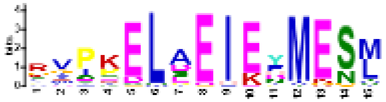	RGPKELAEIEYMESL	4.4e-286	15
